# Silver dressing in the treatment of diabetic foot: A protocol for systematic review and meta-analysis

**DOI:** 10.1097/MD.0000000000024876

**Published:** 2021-02-19

**Authors:** Chunhua Huang, Ruiqi Wang, Zhangren Yan

**Affiliations:** a Affiliated Hospital of Jiangxi University of Traditional Chinese Medicine, Nanchang; b Jiangxi University of Traditional Chinese Medicine Nanchang, Jiangxi Province, China.

**Keywords:** diabetic foot, protocol, silver dressing, systematic review

## Abstract

**Background:**

Diabetic foot (DF) is one of the most common and serious chronic complications of diabetes. At present, there are many dressings used in the treatment of the diabetic foot. Among them, silver dressings are widely used, but the conclusion has not yet been formed. The purpose of this study is to search for relevant studies on the treatment of DF with silver dressings through evidence-based medicine methods and to draw conclusions with higher levels of evidence to provide a basis for the clinical treatment of DF.

**Methods:**

Computer search of databases such as CNKI, SinoMed, VIP, Wanfang, PubMed, Embase, and Cochrane Library. The search time is from the establishment of the database to January 23, 2021. Two researchers will independently select studies, collect data, and assess the methodology quality by the Cochrane risk of bias tool. The meta-analysis will be completed by RevMan 5.3 software.

**Results:**

This systematic review will provide an assessment of the current state of DF, aiming to assess the efficacy of silver dressings for patients with DF.

**Conclusion:**

This systematic review will provide a credible evidence-based for the clinical treatment of DF with silver dressings.

## 1 Introduction

Diabetic foot (DF) is the most serious and common chronic complication of elderly patients with diabetes and in severe cases, the infection can lead to amputation or even death.^[1]^ It is mainly caused by foot (ankle joint or below) infection, ulcer, and (or) deep tissue destruction related to abnormalities of the distal nerves of the lower extremities and various degrees of peripheral vascular disease. Its main clinical manifestations are foot ulcers and gangrene, which are one of the main causes of amputation and disability in diabetic patients. These complications include infection and lower extremity amputation, which have become one of the major causes of disability and death among diabetes patients.^[2]^ With a prevalence rate of 4% to10%,^[3]^ amputation is needed to control the infection to save the lives of patients. Seventy percent of amputations worldwide occur in people with diabetic feet and die after amputation.^[4]^ DF not only affects the physical health of patients but also causes patients to produce anxiety and depression and other unhealthy emotions, bringing a heavy economic burden to the family and society.^[5]^

A wide range of measures have been applied in the management of DF, including debridement, blood glucose control, and infection prevention; however, the clinical effectiveness of these methods is still poor.^[6]^ At present, wound dressing plays an integral part in managing DF. Clinicians have increasingly recognized the value of choosing suitable dressings to manage DF, which can help accelerate wound healing processes, inhibit the propagation of microbes, and improve the wound-healing rate. Some studies have reported that silver dressing is effective in treating DF. Research results show that silver ion dressings can continuously and effectively release silver ions, kill the wound and surrounding bacteria, improve the wound healing environment, and can also hydrate and soften necrotic tissue and clean the wound.^[7]^ Silver ions can bind to negatively charged bacteria, enhance the permeability of the bacterial outer membrane, and induce bacterial apoptosis.^[8]^ The silver dressing has a certain toxic effect on fibroblasts of diabetic patients, which can reduce its cell activity and collagen synthesis, and significantly change cell morphology.^[9]^ Some systematic reviews and meta-analyses have found that silver dressings significantly reduce odor, improve pain-related symptoms, decrease wound exudate, and have a prolonged dressing wear time compared with alternative wound treatments in nonhealing and infected chronic wounds.^[10,11]^

To obtain conclusive evidence of the efficacy of silver dressing in the treatment of diabetic foot, this study used the method of evidence-based medicine meta-analysis to search for clinical randomized controlled trials (RCTs) studies of silver dressing in the treatment of the diabetic foot. To provide evidence-based evidence for clinicians.

## 2 Protocol registration

The system evaluation program will be reported strictly in accordance with the preferred reporting items of the system evaluation and meta-analysis program (PRISMA-P). At the same time, this system evaluation program has been registered on the INPLASY website, the registration number is INPLASY202110112.

## 3 Materials and methods

### 3.1 Literature source

#### 3.1.1 Inclusion criteria

1. The type of literature research is RCT.
2. Study participants: patients diagnosed with diabetic foot, there are no limits to research subjects age, gender, race, condition duration, or intensity. Participants with serious underlying diseases will be excluded.
3. The experimental group used silver dressing care, including various types of silver ion dressings. The control group used other wound dressings, including saline gauze, petrolatum gauze, sterile gauze, wet wound dressing, alginate dressing, foam dressing, etc.
4. Outcome indicators include wound healing rate, granulation tissue appearance time, epithelial formation time, and basic wound healing time, including at least one of them.
5. There was no statistical difference in the baseline comparison of diabetic foot patients.

#### 3.1.2 Exclusion criteria

1. The article does not state that methods such as randomization, comparison, etc., or irregular random allocation methods are used. Case reports, retrospective studies, observational studies, cohort studies, and similar studies will be excluded.
2. The observation group was combined Chinese and Western medicine treatment or silver dressing treatment combined with other treatment methods.
3. The above outcome indicators are not mentioned in the article or the outcome indicators are fuzzy and data cannot be merged.
4. The one with the most recent and most complete data is selected for the repeated publications.
5. Surgical treatment with TCM syndrome differentiation and staging.
6. Research where full text or data cannot be obtained.
7. Animal experiments.

### 3.2 Literature retrieval and data extraction

Search China Knowledge Network (CNKI), China Biomedical Abstracts Database (SinoMed), VIP Database (VIP), Wanfang Database, PubMed, Embase, Cochrane Library, etc. Use corresponding search formulas according to different database requirements. In order to avoid omissions, search scope for including subject words, keywords, or full text. The search time is from the establishment of the database to January 23, 2021. Search terms are: “diabetic foot,” “diabetic feet,” “diabetic foot ulcer,” “foot ulcer,” “Ag,”, “silver dressings,” “silver-releasing dressings,” “silver-impregnated dressings,” “Randomized controlled trial,” “RCT,” The retrieval strategy is shown in Table 1.

**Table 1:** Search strategy used in PubMed database.

Number	Search items
1	diabetic foot
2	diabetic feet
3	diabetic foot ulcer
4	foot ulcer
5	1 or 2–4
6	Ag
7	silver dressings
8	silver-releasing dressings
9	silver-impregnated dressings
10	6 or 7–9
11	Randomized controlled trial
12	RCT
13	11 or 12
14	5 and 10 and 13

### 3.3 Data extraction

After reading the title and abstract, include the required documents, import them into Note Express 3.2.0 for centralized management, and then read the full text and remove them one by one. Two researchers independently screened the literature. When the opinions are inconsistent, the third-party personnel decides to make the basic data extraction table of the literature after the inclusion of the literature is determined, including the author, publication year, research method, research object, diabetes The patient's diagnostic criteria, allocation methods, intervention measures, sample size, duration of treatment, and outcome indicators are adequate.

### 3.4 Risk of bias assessment

Two researchers independently assessed the risk of bias in the included literature with reference to the Cochrane reviewer bias risk assessment tool, including selection bias, implementation bias, measurement bias, follow-up bias, reporting bias, and other source biases for all included documents. Each item will be graded into 3 levels: “high risk,” “low risk,” and “not clear”.^[12]^

### 3.5 Statistical analysis

This research uses the Reviewer Manager 5.3 software provided by Cochrane.^[13]^ Mean difference was used for measurement data, odds ratio was used for classification data, and 95% confidence interval was used as the statistical analysis quantity. If the study includes outcome indicators ≥10, the funnel plot will be used to assess whether publication bias is included in the trial. If there are differences in symmetry or distribution, there will be publication bias or small sample effects.^[14,15]^ Under the test level of α = 0.05, use software to generate a forest map to assess article publication bias. When *P* < 0.05, there is heterogeneity among different studies. Use tests to judge the heterogeneity of the included studies. If there is no statistical heterogeneity among the studies (*P* > .1, *I*^*2*^ < 50%), select the fixed-effects model. If there is statistical heterogeneity between the results of the study (*P* < .1, *I*^2^ > 50%) without clinical heterogeneity, using a random effects model.^[15]^

### 3.6 Sensitivity analysis

The purpose of sensitivity analysis is to eliminate low-quality studies to reduce heterogeneity.^[16]^ By excluding a certain study to observe whether there is a significant change in heterogeneity, the reliability, and stability of the results can be evaluated.

### 3.7 Subgroup analysis

For results with obvious heterogeneity, the source of the heterogeneity should be analyzed.^[17]^ Subgroup analysis can perform heterogeneity tests based on different sources, such as treatment duration, course of the disease, underlying disease, race, gender, age, etc. If there is no clear source of heterogeneity, only descriptive ones can be analyzed.

### 3.8 Ethics and dissemination

Since this is a protocol for systematic review and meta-analysis, all data in this study come from published studies and do not involve patients, so ethical approval is not required. The results of this research will be distributed to peer-reviewed journals and published in relevant conferences.

## 4 Discussion

The defense function of the immune system of diabetic patients is decreased, which makes the foot ulcers of diabetic patients prone to infection, and severe cases can be disabled or even fatal.^[18]^ Some scholars have found that the bacteria in the secretion of diabetic foot wounds are mainly *Staphylococcus aureus*, *Escherichia coli*, *Enterococcus faecalis*, etc., and as the infection deepens, more patients will develop multiple infections and fungal infections, which are difficult to control.^[19]^In recent years, the use of silver ion dressings in various surgical infections has shown good results,^[20]^ and its use in the treatment of DF has also been reported.^[21,22]^ Silver ion dressing is composed of grid structure sodium carboxymethyl cellulose and 1.2% silver ion, which has broad-spectrum antibacterial properties. The silver ions in the dressing are continuously and slowly released and combined with the negative charge on the surface of the bacterial protein, changing the structure of the bacterial body, affecting the replication of genetic material, and killing bacteria, fungi, and other pathogens. But its efficacy has not been evaluated scientifically and systematically. The purpose of this study is to evaluate the clinical efficacy of silver dressing treatment for DF patients. The conclusions drawn by this study may provide evidence-based medical advice for the treatment of DF with silver dressing.

## Author contributions

**Conceptualization:** Zhangren Yan, Chunhua Huang.

**Data curation:** Zhangren Yan, Ruiqi Wang, Chunhua Huang.

**Formal analysis:** Zhangren Yan, Ruiqi Wang.

**Project administration:** Zhangren Yan, Chunhua Huang.

**Supervision:** Zhangren Yan, Chunhua Huang.

**Writing – review & editing:** Zhangren Yan, Chunhua Huang, Ruiqi Wang.
